# Antibacterial potential of a basic phospholipase A_2_ (VRV-PL-VIIIa) from *Daboia russelii pulchella* (Russell’s viper) venom

**DOI:** 10.1186/s40409-015-0014-y

**Published:** 2015-05-28

**Authors:** Shivalingaiah Sudharshan, Bhadrapura Lakkappa Dhananjaya

**Affiliations:** Toxinology Group, Adichunchanagiri Biotechnology and Cancer Research Institute (ABCRI), Balagangadharanatha Nagara, Mandya District, Karnataka India; Toxinology/Toxicology and Drug Discovery Unit, Center for Emerging Technologies (CET), Jain University, Jakksandra Post, Ramanagara, 562112 India

**Keywords:** Snake venom, Bactericidal, Antibiotics, Drug, Human pathogenic bacteria

## Abstract

**Background:**

Microbial/bacterial resistance against antibiotics poses a serious threat to public health. Furthermore, the side effects of these antibiotics have stimulated tremendous interest in developing new molecules from diverse organisms as therapeutic agents. This study evaluates the antibacterial potential of a basic protein, *Vipera russellii* venom phospholipase A_2_ fraction VIIIa (VRV-PL-VIIIa), from *Daboia russelii pulchella* venom against gram-positive and gram-negative bacteria*.*

**Methods:**

The antibacterial potential of VRV-PL-VIIIa in the presence and absence of an inhibitor (p-bromophenacyl bromide) was tested against gram-positive and gram-negative bacteria and the minimum inhibitory concentration was determined by microdilution tests.

**Results:**

VRV-PL-VIIIa demonstrated potent antibacterial activities against all the human pathogenic strains tested. It more effectively inhibited such gram-positive bacteria as *Staphylococcus aureus* and *Bacillus subtilis,* when compared to the gram-negative bacteria *Escherichia coli, Vibrio cholerae, Klebsiella pneumoniae* and *Salmonella paratyphi.* It inhibited bacterial growth at minimum inhibitory concentration values ranging from 11.1 to 19.2 μg/mL. The anti-bacterial potential of VRV-PL-VIIIa was comparable to the standards gentamycin, chlorophenicol and streptomycin. The PLA_2_’s hemolytic and antibacterial activities were strongly correlated. Furthermore, even in the presence of p-bromophenacyl bromide, intense antibacterial activity was observed, suggesting a dissociation or partial overlapping of the bactericidal/antimicrobial domains.

**Conclusion:**

VRV-PL-VIIIa demonstrated potent antibacterial activities against all the human pathogenic strains tested. The study shows that despite a strong correlation between enzymatic and antimicrobial activities of VRV-PL-VIIIa, it may possess additional properties that mimic the bactericidal/membrane permeability-increasing protein. This study encourages further in-depth studies on the molecular mechanisms of antibacterial properties of VRV-PL-VIIIa, which would thereby facilitate development of this protein into a possible therapeutic lead molecule for treating bacterial infections.

## Background

Microbial/bacterial resistance against antibiotics constitutes a therapeutic problem that poses a significant threat to public health [1–4]. The prevalence of bacterial resistance to conventional antibiotics has prompted an intense search for new therapeutic agents from diverse animal origins [5]. Proteins/peptides with potent antimicrobial activities are found in many secretions of organisms, including snakes [5, 6]. Venoms from snakes, particularly Crotalidae, are known to be a rich natural source for the discovery and development of novel antimicrobial agents [6, 7]. Among the various components of snake venom, phospoholipase A_2_ (svPLA_2_) enzyme, apart from its catalytic activity of hydrolyzing the sn-2 ester bond of glycerophospholipids, shows other important toxic/pharmacologic effects that include myonecrosis, neurotoxicity, cardiotoxicity, as well as hemolytic, hemorrhagic, hypertensive, anticoagulant, platelet-aggregation-inhibiting and edema-inducing activities [8, 9]. This array of biological actions may be either dependent or independent of catalytic activities. The svPLA_2_s are also reported to act as antimicrobial agents and are emphasized for their potential as therapeutic lead molecules [6, 7]. Crotapotin, a secretory phospholipase A_2_ isolated from the venom of *Crotalus durissus terrificus*, has been demonstrated to exert antibacterial activity as well as antiviral activity against the human immunodeficiency virus [10–12]. Acidic PLA_2_s, both Asp49 and Lys49 PLA_2_ homologues, have previously been shown to perform bactericidal activity [13–15]. A cationic protein isolated from venom of the inland taipan (*Oxyuranus microlepidotus*) has been demonstrated to selectively and dose-dependently kill the gram-positive bacteria through membrane disruption [16]. Recently, a phospholipase A_2_ from the venom of the saw-scaled viper with novel bactericidal and membrane-damaging activities was characterized [17]. These molecules are shown to be highly attractive due to their biochemical diversity, and broad spectrum of activity against enveloped bacteria, fungi, viruses, protozoa, and parasites [6, 7, 18].

Despite the therapeutic potential of svPLA_2_s as antimicrobial agents, very few with bactericidal/antimicrobial activities have been characterized [6, 18, 19]. Found in India, Russell’s viper (*Daboia russelii*) is a widely distributed snake responsible for potent toxic and lethal effects [20–23]. Despite several reports on its various biological effects, relatively little is known regarding its antimicrobial activity [20–25]. Furthermore, there are no reports available in relation to the antimicrobial activity exhibited by PLA_2_s from the venom of *Daboia russelii pulchella*. A basic PLA_2_, namely *Vipera russellii* venom phospholipase A_2_ fraction VIIIa (VRV-PL-VIIIa), isolated from *Daboia russelii pulchella* venom is reported to provoke various biological effects such as edema, platelet aggregation, pulmonary hemorrhage etc. [26, 27]. In the present work, we evaluate the antibacterial potential of VRV-PL-VIIIa and investigate its possible biochemical mechanism of action. Additionally, this study exemplifies the therapeutic utility of VRV-PL-VIIIa as an antimicrobial agent.

## Methods

Venom of *Daboia russelii pulchella* (Southern region) was purchased from the Irula Co-operative Society Ltd. (India). Agar, beef extract, yeast extract and peptone were bought from Hi Media Private Ltd. (India). The p-Bromophenacyl bromide (p-BPB) and other chemicals used were all of analytical grade and purchased from Sigma Chemicals Ltd. (USA). Authentic pure clinical isolated cultures of human pathogenic bacteria – *Staphylococcus aureus, Bacillus subtilis, Escherichia coli, Salmonella typhi, Vibrio cholerae, Klebsiella pneumoniae* and *Salmonella paratyphi* – were obtained from the Department of Microbiology, Adichunchanagiri Institute of Medical Sciences (AIMS), B.G. Nagara, Karnataka, India. These are all human pathogens that have developed some resistance to common antibiotics particularly in the clinical environment. Bacteria were multiplied in nutrient agar at 36 ± 2 °C. After 2 days, cultures were harvested and prepared at a final concentration of 1 × 10^8^ cfu/mL and used for the *in vitro* inhibition assay.

### Isolation of VRV-PL-VIIIa and chemical modification by p-Bromophenacyl bromide

VRV-PL-VIIIa from the venom of *Daboia ruselii pulchella* was purified until homogeneous as described previously by the method of Kasturi and Gowda [26], with modifications by Srinivasan [27]. The protein concentration was estimated by Lowry’s method. Chemical modification of PLA_2_ by p-Bromophenacyl bromide (p-BPB) was carried out as described by Condrea *et al.* [28]. Briefly, 100 μL of 40 mM p-BPB in acetone was added to 3 mL of PLA_2_ solution (0.5 mg/mL, in 0.05 M Tris–HCl buffer, pH 7.5). The reaction was allowed to proceed for 40 min, and then acidified with glacial acetic acid to pH 4.0 to stop the reaction. Excess of reagent was removed by dialyzing against 0.05 M Tris–HCl buffer pH 7.5.

### Phospholipase A_2_ activity

The phospholipase A_2_ assay was carried out according to the method described by Bhat and Gowda [29]. Phosphatidyl choline (PC) was diluted with petroleum ether (60–80 °C) to obtain a concentration of 1000 nmoles/50 mL. The reaction mixture containing VRV-PL-VIIIa (5 μg) was augmented to 680 μL with water. To the reaction mixture, 200 μL of ether, 100 μL of Tris–HCl buffer (0.05 M, pH 7.5) and 20 μL of CaCl_2_ (0.4 M) were added. The total reaction mixture was incubated at 37 °C for 60 min. After incubation, 0.5 mL of Dole’s mixture (isopropanol:pet ether:1NH_2_SO_4_, 40:10:1) was added, mixed and centrifuged at 1000 rpm for 3 min. To the organic phase, 0.5 mL of CHCl_3_:pet ether (1:5) was added, mixed and centrifuged at 1000 rpm for 3 min. To the upper phase, cobalt reagent [1.35 vol. of triethanolamine increased to 10 mL with solution A (6 g of CO(NO_3_)2.6H_2_O + 0.8 mL glacial acetic acid) and 7 mL of solution B (saturated Na_2_SO_4_)] was added, mixed and centrifuged 1000 rpm for 3 min. The upper organic phase was carefully transferred and 0.75 mL of α-nitroso-β-naphthol reagent (0.4 % α-nitroso-β-naphthol in 96 % ethanol) was added. The intensity of the orange coloration is directly proportional to the amount of cobalt present. After 30 min, 2 mL of ethanol was added to dilute the contents and absorbance was read at 540 nm. The amount of free fatty acid released was estimated using the standard linolenic acid curve. The enzyme activity was expressed as nmoles of fatty acid released/minute/mg of protein.

For inhibition studies, VRV-PL-VIIIa (5 μg) was pre-incubated with or without a different concentration of p-Bromophenacyl bromide (1–6 μm) at 37 °C for 15 min. Appropriate controls were set up and further experiments were performed as described above. The inhibition is expressed as a percentage, considering the activity of venom alone as 100 %.

### Hemolytic activity assay

Hemolytic (direct/indirect) activity of isolated VRV-PL-VIIIa was determined according to the method of Boman and Kaletta [30], using packed human erythrocytes (blood group A). The human erythrocytes used for the study were sourced from previously published work, which had ethical approval from the ethics committee of the University of Mysore (UOM) for the withdrawal of blood [23]. The direct and indirect hemolytic assays were carried out using washed erythrocytes. For the direct hemolytic assay, the packed erythrocytes (1 mL) were suspended in nine volumes of phosphate-buffered saline (PBS), which formed the stock. The stock (1 mL) was incubated with various concentrations of isolated VRV-PL-VIIIa (0–5 μg) for 30 min at 37 °C. For the indirect hemolytic assay, stock was prepared by mixing packed erythrocytes (1 mL), egg yolk (1 mL) and phosphate-buffered saline (8 mL). One milliliter of suspension from stock was incubated with various concentrations of isolated VRV-PL-VIIIa (0–6 μg) for 30 min at 37 °C. The reaction was terminated by adding 10 mL of ice-cold PBS and then centrifuged at 4 °C and 800 g. The amount of hemoglobin released in the supernatant was measured at 540 nm. One milliliter of stock erythrocytes with 10 mL ice-cold PBS alone was defined as constituting 0 % lysis.

For inhibition studies, VRV-PL-VIIIa (6 μg) was pre-incubated with or without a different concentration of p-Bromophenacyl bromide (1–6 μM) at 37 °C for 15 min. Appropriate controls were set up and further experiments were accomplished as described above. The inhibition is expressed as a percentage, considering the activity of venom alone as 100 %.

### Bactericidal activity of VRV-PL-VIIIa

Bactericidal activity was evaluated by the well diffusion method on nutrient agar medium [31]. This was confirmed by the inhibitory effect on bacterial growth as reflected by the inhibition zone, compared to that of known antibiotics such as gentamicin (G), chloramphenicol (Cp) and streptomycin (Sm) at 30 μg/mL. The sterile nutrient agar medium (20 mL) in petri dishes was uniformly smeared using sterile cotton swabs with pure test cultures of the human pathogenic bacteria *S. aureus, B. subtilis, E. coli, S. typhi, V. cholerae, K. pneumoniae* and *S. paratyphi*. The nutrient agar media was prepared by dissolving 0.3 % beef extract, 0.3 % yeast extract, 0.5 % peptone, 0.5 % NaCl and 1.5 % agar in 1:l of distilled water and 0.2 % methanol (v/v). The wells of 5 mm diameter were made using a sterile cork borer in each petri dish and the isolated VRV-PL-VIIIa (0-12 μg) pre-incubated independently with or without p-Bromophenacyl bromide (15 μM) was added; a blank well loaded without test compound was considered the control. For each treatment, ten replicates were prepared. The plates were incubated at 37 °C for 24 h and the resulting zone of inhibition was measured by comparing the control and the standard antibiotics.

For inhibition studies, VRV-PL-VIIIa (12 μg) was pre-incubated with or without a different concentration of p-Bromophenacyl bromide (1–6 μM) at 37 °C for 15 min and antimicrobial activity was assessed as described above with appropriate controls. The inhibition is expressed as a percentage, considering the activity of venom alone as 100 %.

### Determination of minimum inhibitory concentration (MIC)

The minimum inhibitory concentration of the isolated VRV-PL-VIIIa and the antibiotics used were determined by serial dilution in the nutrient agar, with concentrations ranging from 2–20 μg/mL. The inoculum was prepared overnight from fresh broth culture in nutrient broth and plates were incubated for 24 h at 37 °C. MIC was recorded as the lowest VRV-PL-VIIIa at which the antibiotic concentrations demonstrated no visible growth in the broth [32].

### Statistical analysis

Statistical analysis was done using the software SPSS (Windows version 10.0.1; SPSS Inc., USA) employing a one-way Student’s *t* test; p < 0.05 was considered as statistically significant when compared with relevant controls. All results are presented as mean ± SD.

## Results and discussion

The snake venom PLA_2_s (svPLA_2_s), apart from their well known toxic and lethal effects, are also known to be of therapeutic utility, particularly as bactericidal/antibacterial agents [6, 19]. In a previous study, it was shown that the crude Russell’s viper venom from India exhibited strong antibacterial actions [25]. However, the principal component responsible for it was unexplored. In the present work, we evaluate the basic PLA_2_ (VRV-PL-VIIIa) of *Daboia russelii pulchella* venom for its antibacterial activity on different human pathogenic bacteria [26, 27].

**Fig. 1:**
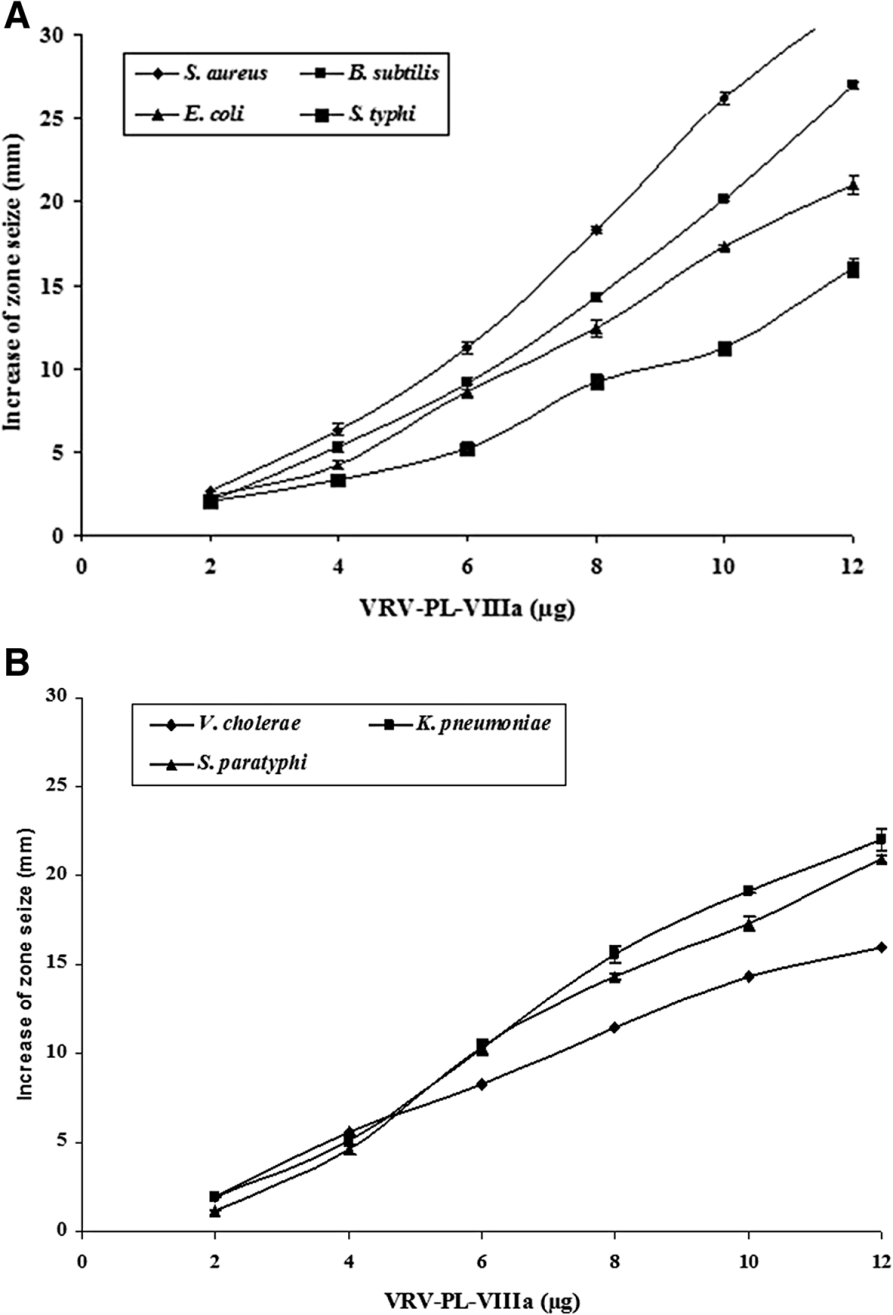
**a** Dose-dependent bactericidal activity of VRV-PL-VIIIa against different human pathogenic strains – *S. aureus, E. coli, B. subtilis, S. typhi* – in agar diffusion assays. The diameter of the clear zone was measured and plotted after subtracting the diameter of the well (5 mm). Results are mean ± SD for three independent assays, each performed in triplicate. **b** Dose-dependent bactericidal activity of VRV-PL-VIIIa against different human pathogenic strains – *V. cholerae, K. pneumonia, S. paratyphi –* in agar diffusion assays. The diameter of the clear zone was measured and plotted after subtracting the diameter of the well (5 mm). Results are mean ± SD for three independent assays, each performed in triplicate

When tested, VRV-PL-VIIIa (0–12 μg/mL) showed a broad spectrum of highly significant antibacterial activities by producing a clear zone of inhibition that was dose-dependent in the range of 17–30 mm (Fig. 1 – a and b) (Table 1). Interestingly, it showed more significant inhibition of gram-positive bacteria including *S. aureus* and *B. subtilis* (in the range of 27–32 mm) in relation to such gram-negative species as *E. coli*, *S. typhi, V. cholerae, K. pneumoniae* and *S. paratyphi* (in the range of 16–22 mm) (Table 1). Furthermore, it was interesting to note that VRV-PL-VIIIa exhibited similar or greater antibacterial activities than those of the standards gentamicin, chlorophenicol and streptomycin (which was in the range of 16–20 mm) (Table 1). When VRV-PL-VIIIa was tested via the agar dilution assay for determining minimum inhibitory concentration (MIC), it significantly inhibited the bacterial growth with MIC values ranging from 11.2 to 20 μg/mL, when compared to standard antibiotics whose range was between 18 and 24 μg/mL (Table 2). Thus, VRV-PL-VIIIa was as potent as standard antibiotics in inhibiting the growth of bacterial strains.

**Table 1:** Antibacterial activity of VRV-PL-VIIIa and standard antibiotics

Microorganisms	Diameter of inhibition zone (mm)			
	VRV-PL-VIIIa	G^a^	Cp^a^	Sm^a^
Gram-positive				
*Staphylococcus aureus*	32 ± 1	18 ± 1	23 ± 2	26 ± 2
*Bacillus subtilis*	27 ± 4	18 ± 2	19 ± 2	28 ± 3
Gram-negative				
*Escherichia coli*	21 ± 2	18 ± 2	18 ± 2	21 ± 2
*Salmonella typhi*	16 ± 1	17 ± 2	18 ± 1	18 ± 1
*Vibrio cholerae*	16 ± 3	16 ± 2	19 ± 2	19 ± 2
*Klebsiella pneumoniae*	22 ± 2	18 ± 2	18 ± 1	21 ± 3
*Salmonella paratyphi*	21 ± 6	19 ± 2	18 ± 2	20 ± 2

The results are mean ± SD (*n* = 6)

Standard antibiotics^a^ *G* gentamicin, *Cp* chloramphenicol, *Sm* streptomycin

**Table 2:** Minimum inhibitory concentration (MIC) of VRV-PL-VIIIa and standard antibiotics by serial dilution method

Microorganisms	MIC (μg/mL)			
	VRV-PL-VIIIa	G	Cp	Sm
Gram-positive				
*Staphylococcus aureus*	15.3 ± 1	20.8 ± 1	14.4 ± 2	13.6 ± 1
*Bacillus subtilis*	11.1 ± 3	20.8 ± 3	14.4 ± 1	16.6 ± 1
Gram-negative				
*Escherichia coli*	16.3 ± 2	23.8 ± 1	14.4 ± 2	14.6 ± 1
*Salmonella typhi*	17.2 ± 2	18.8 ± 1	17.4 ± 2	13.6 ± 1
*Vibrio cholerae*	17.3 ± 2	19.8 ± 3	14.4 ± 1	19.6 ± 1
*Klebsiella pneumoniae*	19.2 ± 1	20.8 ± 1	14.4 ± 2	13.6 ± 1
*Salmonella paratyphi*	17.3 ± 2	23.8 ± 1	14.4 ± 2	14.6 ± 1

The results are mean ± SD (*n* = 6)

*G* gentamicin, *Cp* chloramphenicol, *Sm* streptomycin

**Fig. 2:**
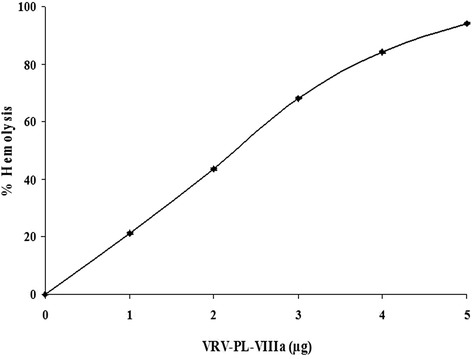
Dose-dependent indirect hemolytic activity of VRV-PL-VIIIa. VRV-PL-VIIIa (0–5 μg) in 100 μL of phosphate-buffered saline (PBS) was incubated with erythrocytes, egg yolk and PBS (1:1:8 v/v) for 10 min at 37 °C. The released hemoglobin in the supernatant was measured by reading absorbance at 540 nm. The results are expressed as ± S.E.M. (*n* = 4)

A strong correlation is usually found between the PLA_2_’s hemolytic and antibacterial activities [6, 19, 25]. The VRV-PL-VIIIa also showed a potent hemolytic (indirect) activity that is usually associated with svPLA_2_s. It was found that VRV-PL-VIIIa produces a dose-dependent hemolysis of blood cells and at 5 μg concentration provoked 100 % hemolysis (Fig. 2). From these data it may be concluded that the antibacterial effects of VRV-PL-VIIIa are dependent upon catalytic activity, i.e., an enzymatic membrane degradation effect that is usually observed in sPLA_2_s [6, 33]. The correlation between PLA_2_, hemolytic and antibacterial activities indicates that the catalytically activity of PLA_2_ is principally involved in bactericidal/antibacterial activities [6, 19, 25]. However, despite the existence of another mechanism, namely svPLA_2_ isolated from *Bothrops asper* (also classified within group IIA) venom, which was shown to directly kill both gram-positive and gram-negative bacteria [13].

**Fig. 3:**
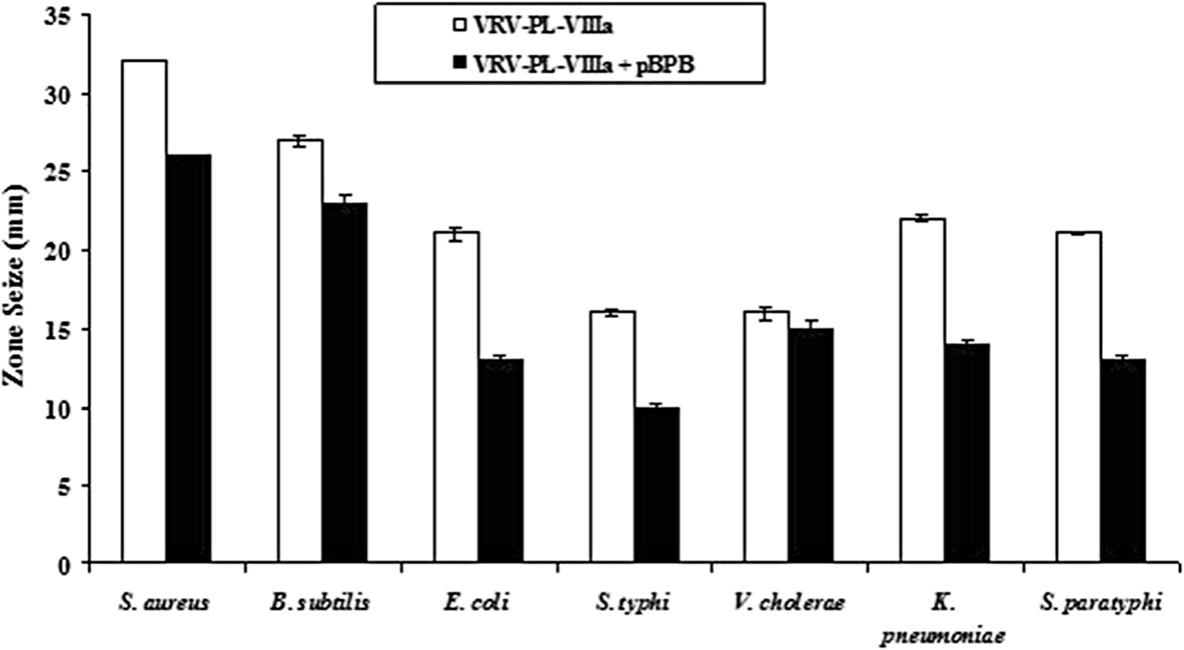
Bactericidal activity against different human pathogenic strains of VRV-PL-VIIIa pre-incubated with or without p-Bromophenacyl bromide. VRV-PL-VIIIa (12 μg/mL) was pre-incubated with or without a different concentration of p-Bromophenacyl bromide (6 μM) at 37 °C for 15 min and bactericidal activity was estimated in agar diffusion assay. The diameter of the clear zone was measured and plotted after subtracting the diameter of the well (5 mm). Results are expressed as mean ± SD for three independent assays, each performed in triplicate

Furthermore, a toxin from *B. asper* (myotoxin II, a catalytically inactive Lys49 PLA_2_) exhibited a bactericidal mechanism that was independent of catalytic activity [13, 6, 19]. Additional in-depth studies showed that a short sequence of the protein, corresponding to residues 115–129 of its cytolytic C-terminal region, was responsible for its bactericidal activity, emphasizing the fact that bactericidal activity is not associated with enzymatic activities [6, 13, 19]. Similarly, the study found that when VRV-PL-VIIIa was pre-incubated with p-BPB, an inhibitor of svPLA_2_ enzymatic activity, both enzymatic and antibacterial activities were inhibited (Fig. 3 and Table 3) [34]. However, it should be noted that there was incomplete abolition of antibacterial activity (Table 3), even though the enzymatic activity was completely abolished (Table 4). This suggests a dissociation between enzymatic activity and bactericidal/antibacterial activity of VRV-PL-VIIIa. It is usually observed that the potent bactericidal activity of sPLA_2_s is accomplished by binding to anionic surfaces along with the enzymatic degradation of phospholipids in the target membranes, i.e., preferentially in the case of gram-positive species. However, the bactericidal activity against gram-negatives is known to require a synergistic action of bactericidal/permeability-increasing protein (BPI), and is also equally dependent on enzymatic-based membrane degradation [13, 6, 17, 19].

**Table 3:** Antibacterial activity of VRV-PL-VIIIa with or without p-Bromophenacyl bromide (p-BPB)

Microorganisms	Diameter of inhibition zone (mm)	
	VRV-PL-VIIIa	VRV-PL-VIIIa + p-BPB
Gram-positive		
*Staphylococcus aureus*	32 ± 1	23 ± 3
*Bacillus subtilis*	27 ± 4	19 ± 1
Gram-negative		
*Escherichia coli*	21 ± 2	13 ± 1
*Salmonella typhi*	16 ± 1	10 ± 3
*Vibrio cholerae*	16 ± 3	15 ± 2
*Klebsiella pneumoniae*	22 ± 2	14 ± 2
*Salmonella paratyphi*	21 ± 6	13 ± 3

The results are expressed as mean ± SD (*n* = 6)

**Table 4:** Phospholipase A_2_ activity of VRV-PL-VIIIa with or without p-Bromophenacyl bromide (p-BPB)

Specific activity		
Enzymatic activity	VRV-PL-VIIIa	VRV-PL-VIIIa + p-BPB
PLA_2_^a^	93.4 ± 3.1	3.2 ± 0.8

Values are presented as mean ± SD (*n* = 6)

^a^Specific activity is expressed in terms of fatty acid released in nmoles/minute/mg of protein

It has been demonstrated that, in addition to cationic properties of PLA_2_ molecules, the polyanionic properties of lipoteichoic acids from bacterial cell wall promote the attack of membrane phospholipids by svPLA_2_s. Therefore, the action mode of svPLAs depends on the type of bacteria species involved (gram-positive or negative). Recently, an association was shown between antibacterial and enzymatic activity of an antibacterial PLA_2_ (EcTx-I) purified from *Echis carinatus* venom [6]. However, the present study found that the bactericidal activity of VRV-PL-VIIIa is partially independent of catalytic activity and antibacterial activities (Tables 3 and 4), which is supported by the homogenous nature of the protein with no other associated enzymatic activities (such as L-amino-oxidase, proteases etc.) in the preparation (data not shown). Therefore, the VRV-PL-VIIIa bactericidal mechanism may include “fatal depolarization” of the bacterial membrane, creation of physical holes in the membrane, scrambling of normal distribution of lipids between the bilayer leaflets, damage of critical intracellular targets after internalization of the peptide, and also inhibition of macromolecular biosynthesis as observed in many svPLA(_2_)s and/or interaction with specific vital components inside the bacteria [6, 35].

It was reported that PLA_2_s purified from *Agkistrodon piscivorus piscivorus* rely on a membrane-permeabilizing mechanism to exert their bactericidal effects [36]. A recent study showed the presence of a large number of PLA_2_-sensitive phospholipid domains/composition, rather than only the phosphatidylcholine (PC) content of a particular membrane that determines the extent of membrane damage by a particular venom PLA_2_ enzyme [37]. This might be one of the reasons for the differential inhibitory potency of VRV-PL-VIIIa on various bacterial species. Therefore, it may be concluded that the isolated VRV-PL-VIIIa phospholipase A_2_, will manifest its antimicrobial activity not only by acting upon the membrane through its enzymatic activity, but also by other mechanisms as discussed above, independently of its catalytic activities. Further in-depth studies on molecular mechanism of action of VRV-PL-VIIIa antibacterial activity would be of interest to develop this as a therapeutic lead molecule for application.

## Conclusion

This study shows that VRV-PL-VIIIa – a PLA_2_ from *Daboia russelii pulchella* venom – presents potent antibacterial activity. Significant antibacterial activity is observed, even in presence of an inhibitor of PLA_2_ enzymatic activity (p-BPB), suggesting a dissociation or partial overlapping of the bactericidal/antimicrobial domains of the enzyme. This study demonstrates that although there is a strong correlation between enzymatic and antimicrobial activities of VRV-PL-VIIIa, it may also possess other properties that mimic the bactericidal/membrane permeability-increasing protein. These results should encourage further in-depth studies on molecular mechanisms of anti-bacterial properties of VRV-PL-VIIIa, which would thereby facilitate its development into a therapeutic lead molecule for treating bacterial infections.

### Ethics committee approval

The use of human erythrocytes was approved by the Ethics Committee of the University of Mysore (UOM).
